# Transarterial chemoembolization with 125 I seed insertion for unresectable hepatocellular carcinoma: a meta‑analysis

**DOI:** 10.20452/wiitm.2025.17931

**Published:** 2025-03-24

**Authors:** Rui Zhu, Kui Mao, Xin‑Zhi Lu, You‑Qing Wang, Qian‑Qian Li, Zi Ye

**Affiliations:** Zhejiang Chinese Medical University, Hangzhou, China; Department of Interventional Radiology, The First People’s Hospital of Linhai City, China; Department of Vascular Surgery, The First People’s Hospital of Linhai City, China; Department of General Surgery, The First People’s Hospital of Linhai City, China; General Practice Department, The First People’s Hospital of Linhai City, China

**Keywords:** hepatocellular cancer, iodine‑125, meta analysis, transarterial chemoembolization, treatment

## Abstract

**INTRODUCTION:**

Transarterial chemoembolization (TACE) is frequently used to treat patients with hepa‑ tocellular cancer (HCC) who are not eligible for surgery. The efficacy of TACE treatment can be improved by percutaneous insertion of 125I seeds after the procedure.

**AIM:**

This meta‑analysis aimed to assess the relative safety and efficacy of TACE with 125I seed insertion (TACE‑I) as compared with TACE alone for the management of inoperable HCC.

**MATERIALS AND METHODS:**

The PubMed, Cochrane Library, and Wanfang databases were searched for relevant studies published since the database inception through October 2024. The primary study outcome was objective response rate (ORR), while secondary outcomes comprised disease control rate (DCR), progression‑free survival (PFS), overall survival (OS), and adverse event incidence.

**RESULTS:**

This meta‑analysis ultimately included 5 articles, all of which were published in China. In all these studies, TACE‑I outperformed TACE alone with respect to patient ORR (P <0.001), PFS (P <0.001), and OS (P <0.001). DCR values were similar in both groups (P = 0.77), as were the rates of adverse events, including fever (P = 0.75), vomiting (P = 0.83), and myelosuppression (P = 0.23). The only outcome exhibiting significant heterogeneity was OS (I2 = 73%). Based on the Egger test, the end points affected by publication bias were ORR, DCR, and OS (P = 0.01, P = 0.03, and P = 0.04, respectively).

**CONCLUSIONS:**

In patients with inoperable HCC, TACE‑I is associated with significantly better efficacy and longer survival than TACE alone, and has a good safety profile.

## INTRODUCTION

Hepatocellular cancer (HCC) is a frequently diagnosed liver malignancy.^1^^,^^2^^,^^3^ The optimal approaches to HCC treatment include surgical resection of the tumor or liver transplantation. Unfortunately, over 60% of patients are ineligible for surgical resection,^4^ and a limited availability of liver grafts often serves as a barrier to transplantation.^5^

Transarterial chemoembolization (TACE) is a procedure that is recommended for patients with Barcelona Clinic Liver Cancer (BCLC) stage B HCC as well as for some individuals with BCLC stage A HCC who are not eligible for surgical resection.^6^^,^^7^ TACE is often used in combination with percutaneous ablation to facilitate further disruption of target tumor tissues.^5^^,^^6^^,^^7^ However, factors such as location of the target tumor or the heat sink effect may render some patients un‑ suitable for percutaneous ablation.^4^ In these cases, percutaneous ^125^I seed insertion is a promising alternative treatment strategy.^4^ ^125^I seeds are synthetic radionuclides often used to treat HCC. They emit X‑rays and γ‑rays that cause DNA dam‑ age in the nearby tumor cells and induce free radical production, ultimately leading to tumor cell death.^8^ Percutaneous insertion of ^125^I seeds has been used to treat a range of solid tumor types.^9^ However, the efficacy of combining TACE with 125I seed insertion (TACE‑I) for the management of inoperable HCC remains poorly understood.

**TABLE 1:** General characteristics of the included studies

Study	Year of publication	Country / area	Design	NOS, points
Chen et al^11^	2020	China	Retrospective	8
Gao et al^12^	2022	China	Retrospective	8
Li et al^13^	2016	China	Retrospective	7
Wang et al^14^	2023	China	Retrospective	8
Wang et al^15^	2024	China	Retrospective	8

Abbreviations: NOS, Newcastle‑Ottawa Scale

## AIM 

This meta‑analysis sought to compare the relative safety and efficacy of TACE‑I vs TACE alone for the treatment of patients with HCC.

## MATERIALS AND METHODS 

Study selection The Preferred Reporting Items for Systematic Reviews and Meta‑Analyses were used to guide this meta‑analysis. The study was registered at INPLASY.COM (No. INPLASY2024110047).

Relevant articles published as of October 2024 in the PubMed, Cochrane Library, and Wanfang databases were identified using the following search strategy: (((iodine‑125) OR (I^125^)) AND ((transarterial chemoembolization) OR (TACE))) AND ((hepatocellular carcinoma) OR (HCC)). The EndNote X7 software (Thomson Corporation, Stanford, Connecticut, United States) was used to aid in the study se‑ lection process.

The following eligibility criteria were applied:

1) types of article: original articles; 2) diseases: inoperable HCC; 3) types of treatment: TACE‑I and TACE alone; 4) language of publication: English.

Studies were excluded if they 1) involved nonhu‑ man subjects; 2) focused on TACE combined with ^125^I seed stent placement in HCC patients with an obstructed portal vein; 3) had a sample size of less than 20 participants.

### Data extraction

The studies were selected by 2 investigators (RZ and KM), who extracted all data independently of one another, with any disagreements being resolved by a third investigator (ZY). Data extracted from the studies included the name of the first author, year of publication, country of origin, study design, baseline patient data, baseline HCC data, and treatment‑related outcomes. Objective response rate (ORR) was the primary outcome in this meta‑analysis, whereas secondary outcomes included disease control rate (DCR), progression‑free survival (PFS), overall survival (OS), and adverse event incidence. ORRs were calculated as the overall rate of complete and partial responses, while DCRs were calculated as the overall ORRs and stable disease rates. The modified Response Evaluation Criteria in Solid Tumors were used to assess the response to treatment.^10^

### Quality analysis

The quality of studies was assessed with the Newcastle‑Ottawa scale (NOS), assigning 4, 3, and 2 points to selection, exposure, and comparability criteria, respectively. A NOS score greater than or equal to 7 was considered indicative of high study quality.

### Statistical analysis

RevMan v5.3 (The Cochrane Collaboration, Copenhagen, Denmark) and Stata 12.0 (Stata Corporation LLC, College Station, Texas, United States) software were used to implement this meta‑analysis. Dichotomous variables were pooled as odds ratios (ORs) with 95% CIs. OS and PFS were compared using hazard ratios (HRs). The Q test and I2 statistic were used to evaluate het‑ erogeneity, with random‑effect models being used in cases of significant heterogeneity (I2 >50%), whereas all other end points were analyzed with a fixed effect model. Leave‑one‑out sensitivity analyses were used to probe for sources of heterogeneity. The Egger test was used to evaluate potential publication bias. A P value below 0.05 was used to define statistical significance.

### Ethics statement

As this was a meta‑analysis of published data, it did not require additional ethical approval or written informed consent from the patients.

## RESULTS

### Study selection

**FIGURE 1:**
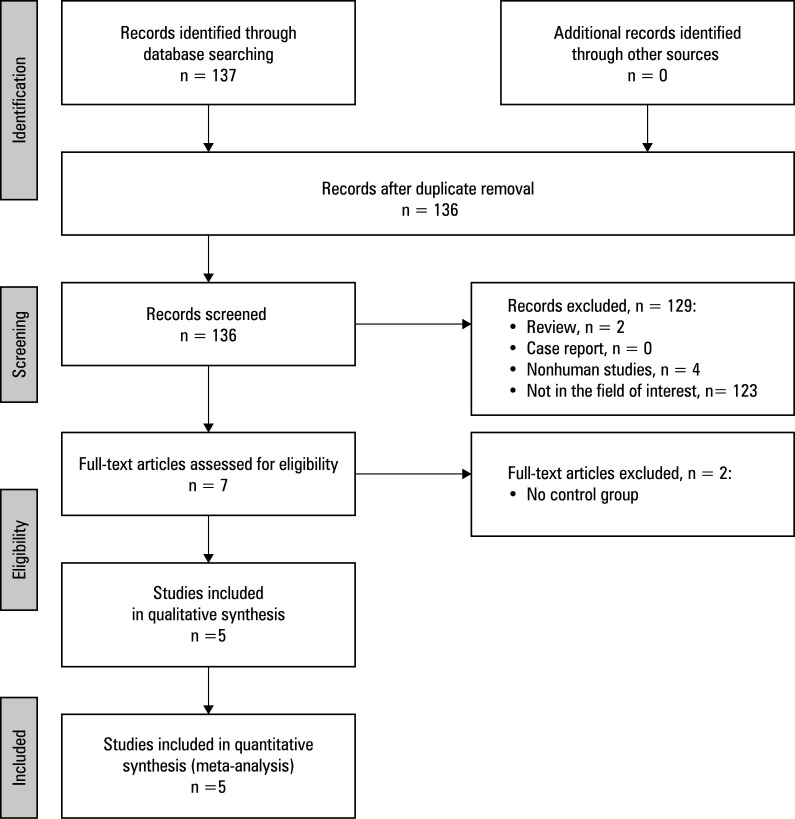
Meta‑analysis flow chart

Initially, a total of 137 articles were retrieved using the selection strategy specified above. Of those, 5 studies met the in‑ clusion criteria.^11^^,^^12^^,^^13^^,^^14^^,^^15^ All of them were retrospective analyses conducted in China TABLE 1 . They were published between 2016 and 2024, and had NOS scores ranging from 7 to 8. The study selection process is detailed in FIGURE 1.

These 5 studies included 204 and 218 patients who underwent TACE‑I and TACE treatment, respectively TABLE 2 . There were no significant differences between the groups with respect to age, sex, tumor staging, liver function, or number of tumors. One of the analyzed studies evaluated multiple HCC tumors.^15^Treatment‑related outcome data are presented in TABLE 3.

### Overall response rate 

**FIGURE 2:**
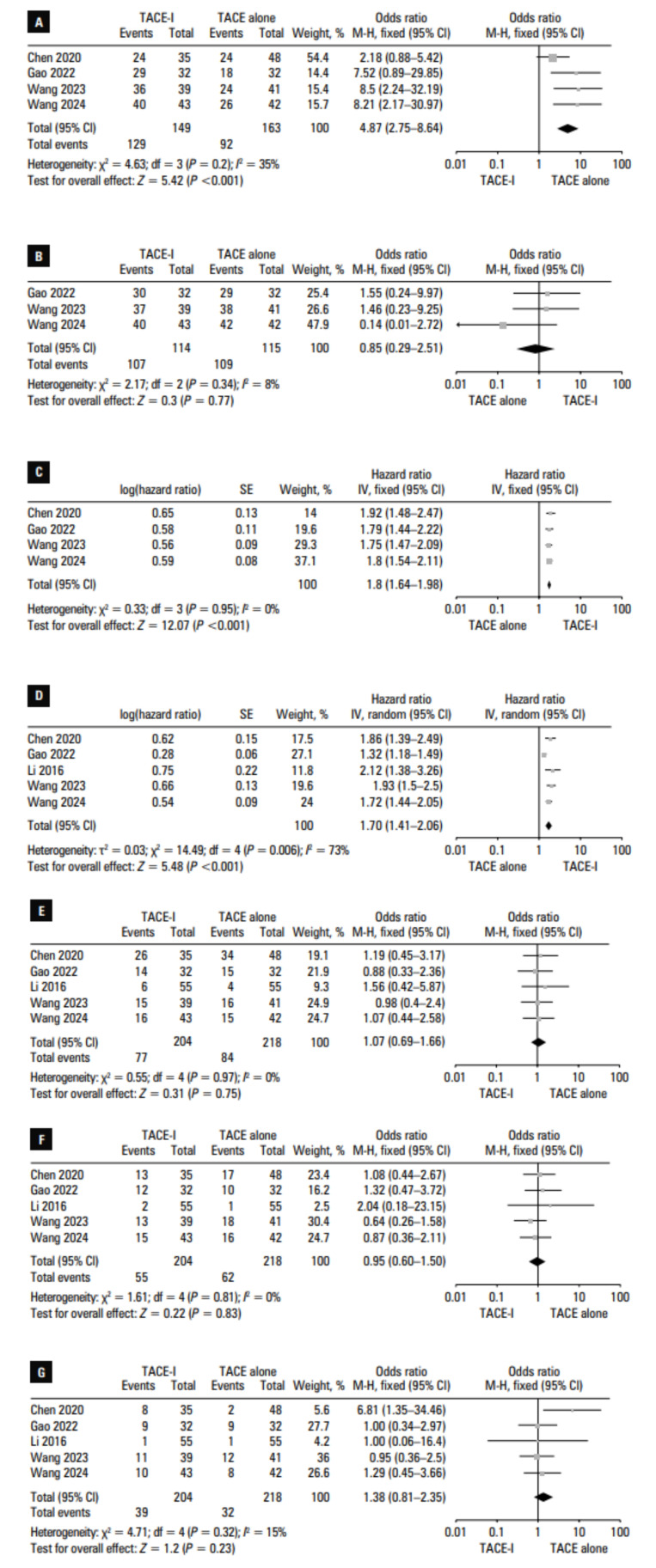
Pooled results for objective response rate (A), disease control rate (B), progression‑free survival (C), overall survival (D), fever rate (E), vomiting rate (F), and myelosuppression rate (G) in the TACE‑I and TACE alone groups

Abbreviations: df, degree of freedom; IV, instrumental variable; M-H, Mantel–Haenszel test, others, see TABLE 2

ORRs were reported in 4 of the included studies.^11^^,^^12^^,^^14^^,^^15^ A significantly higher pooled ORR was noted in the TACE‑I vs the TACE group (86.6% vs 56.4%; OR, 4.87; 95% CI, 2.75–8.64;P <0.001; FIGURE 2A). This end point did not exhibit significant heterogeneity (I2 = 35%).

### Disease control rate 

DCRs were reported in 3 of the included studies.12,14,15 Pooled DCRs were com‑ parable in the TACE‑I and TACE groups (93.9% vs 94.8%; OR, 0.85; 95% CI, 0.29–2.51; P = 0.77; FIGURE 2B). This end point did not exhibit significant heterogeneity (I2 = 8%).

### Progression‑free survival

PFS rates were compared between the TACE‑I and TACE groups in 4 studies.^11^^,^^12^^,^^14^^,^^15^ Based on the pooled log‑transformed HR, the PFS of the patients treated with TACE‑I was significantly longer than that of the patients treated with TACE alone (HR, 1.66; 95% CI, 1.46–1.89; P <0.001; FIGURE 2C). This end point did not exhibit significant heterogeneity (I^2^ = 0%).

### Overall survival

OS data were reported in all 5 studies. The pooled log‑transformed HR values indicated that OS was longer in the TACE‑I than in the TACE group (HR, 1.7; 95% CI, 1.41–2.06;P <0.001; FIGURE 2D). This end point was associated with significant heterogeneity (I2 = 73%) driven by the study by Gao et al.^12^ When this study was excluded from the analysis, heterogeneity was no longer observed (I2 = 0%), and the OS of the patients treated with TACE‑I was still found to be longer than that of the patients treated with TACE alone (P <0.001).

### Fever

Fever rates were reported in all 5 studies, and the pooled rates were similar in the TACE‑I and TACE groups (37.7% vs 38.5%; OR, 1.07; 95% CI, 0.69–1.66; P = 0.75; FIGURE 2E). This end point did not exhibit any heterogeneity (I2 = 0%).

### Vomiting

Rates of vomiting were presented in all 5 studies, and were comparable in the TACE‑I and TACE groups (30% vs 28.4%; OR, 0.95; 95% CI, 0.6–1.5; P = 0.83; FIGURE 2F). No heterogeneity was observed for this end point (I2 = 0%).

### Myelosuppression

Myelosuppression rates were reported in all analyzed studies. Similar pooled rates of myelosuppression were observed in the TACE‑I and TACE groups (19.1% vs 14.7%; OR, 1.38; 95% CI, 0.81–2.35; P = 0.23; FIGURE 2G). This end point did not exhibit significant heterogeneity (I2 = 15%).

### Publication bias 

Based on the results of the Egger test, publication bias was found for ORR, DCR, and OS (P = 0.01, P = 0.03, and P = 0.04, respectively). The remaining end points did not exhibit significant publication bias.

## DISCUSSION 

TACE is the most widely used interventional treatment for inoperable HCC,^16^^,^^17^^,^^18^ and it serves as the first‑line management approach in this patient population.^16^^,^^17^^,^^18^ To achieve greater clinical efficacy, TACE is often combined with other therapeutic approaches, such as immunotherapy, ablation, or 125I seed insertion.^4^^,^^5^^,^^6^^,^^19^

In this meta‑analysis, treatment responses, survival outcomes, and adverse event rates were used to evaluate the relative safety and efficacy of TACE‑I vs TACE alone for the treatment of inoperable HCC. Based on our findings, the use of TACE‑I resulted in significantly better ORR, as compared with TACE alone, with the TACE‑I group exhibiting a pooled ORR as high as 86.6%. Studies of patients with inoperable HCC who underwent drug‑eluting bead (DEB)‑TACE reported similarly high ORRs, ranging between 72% and 90.1%.^20^^,^^21^^,^^22^ The short-term efficacy of TACE‑I may thus be similar to that of DEB‑TACE in these HCC patient populations. However, in tumors with poor blood supply, DEB‑TACE may fail to achieve complete tumor embolization, whereas ^125^I seed insertion can help eliminate any residual tumor cells after TACE.^23^

**TABLE 2:** Baseline data of the participants

Author	Group	Patients, n	Mean age, y	Sex, M/F	Mean tumor size	BCLC stage	Child‑Pugh classes	Number of tumors
Chen et al^11^	TACE alone	48	59.6	38/10	<3 cm, n = 13; ≥3 cm, n = 35	A–C	A–B	Single, n = 24; multiple, n = 24
	TACE‑I	35	58.1	26/9	<3 cm, n = 14; ≥3cm, n = 21	A–C	A–B	Single, n = 16; multiple, n = 19
Gao et al^12^	TACE alone	32	62.1	26/6	5.8 cm	A–C	A–B	Single, n = 19; multiple, n = 12
	TACE‑I	32	62.7	26/6	5.5 cm	A–C	A–B	Single, n = 24; multiple, n = 8
Li et al^13^	TACE alone	55	48.5	47/8	N/A	A–C	A–B	Single, n = 18; multiple, n = 37
	TACE‑I	55	48.4	48/7	N/A	A–C	A–B	Single, n = 20; multiple, n = 35
Wang et al^14^	TACE alone	41	62.4	33/8	5.7 cm	A–B	A–B	Single, n = 25; multiple, n = 16
	TACE‑I	39	62	29/10	5.2 cm	A–B	A–B	Single, n = 22; multiple, n = 17
Wang et al^15^	TACE alone	42	60.8	32/10	2.9 cm	A–B	A–B	Multiple (all patients)
	TACE‑I	43	58.5	39/4	3.1 cm	A–B	A–B	Multiple (all patients)

Abbreviations: BCLC, Barcelona Clinic Liver Cancer; F, female; M, male; N/A, not available; TACE, transarterial chemoembolization, TACE‑I, TACE with ^125^I seed insertion

**TABLE 3:** Treatment characteristics

Study	Groups	Objective response rate, %	Disease control rate, %	Progression-free survival, mo	Overall survival, mo
Chen et al^11^	TACE alone	50	N/A	8	23
	TACE-I	68.6	N/A	16	42
Gao et al^12^	TACE alone	59.4	90.7	5	18
	TACE-I	90.7	93.8	11	22
Li et al^13^	TACE alone	N/A	N/A	N/A	18
	TACE-I	N/A	N/A	N/A	30
Wang et al^14^	TACE alone	58.5	92.7	2	6
	TACE-I	92.3	97.4	4	10
Wang et al^15^	TACE alone	61.9	100	7	15
	TACE-I	93	93	13	23

Abbreviations: see TABLE 2

While this meta‑analysis showed that treatment with TACE‑I was associated with a better ORR, the pooled DCRs for TACE‑I and TACE alone were similar. This may suggest that TACE alone is effective in achieving local disease control in HCC, and that 125I seeds can facilitate further tumor cell destruction but fail to provide protection against tumor progression, particularly with respect to extra‑hepatic progression. 

The presented results demonstrate a clear link between TACE‑I and better OS and PFS, as compared with TACE alone. As ^125^I seeds emit low‑dose radiation, they can damage cycle‑sensitive cells so that they are ultimately eliminated, leading to shifts in overall tumor cell distributions that may render these tumors more chemosensitive, conferring better long‑term treatment efficacy.^12^ However, significant heterogeneity was observed for the OS end point, so the reliability of these results is uncertain and there is a clear need for a series of prospective randomized controlled trials aimed at validating these findings.

Here, ^125^I seed insertion was not associated with an increase in adverse event risk. This positive outcome may be attributable to the appropriate use of the treatment planning system to select optimal ^125^I seed number and distribution in a manner intended to minimize radiation‑induced damage to peritumoral healthy tissues. 

There are some limitations to this meta‑analysis. Firstly, all of the included studies had a retrospective design, which may be associated with heterogeneity in outcome reporting and selection bias. In addition, relatively few studies were included in the pooled analyses, and all of them were from China. As a consequence, these results may only reflect the clinical situation in China, which is a country with the largest number of HCC patients globally. Nevertheless, our findings support the efficacy of TACE‑I as an approach to HCC management.

## CONCLUSIONS 

In summary, the results of this meta‑analysis demonstrate that, relative to TACE alone, TACE‑I is associated with significantly better efficacy and prolonged survival when used to manage inoperable HCC, and it does not entail any additional safety risks.
